# A preliminary analysis of the inflammatory protein landscape in the CSF of mid- to late-stage Parkinson’s disease: associations with motor severity and subtypes

**DOI:** 10.1186/s12883-026-05109-8

**Published:** 2026-07-01

**Authors:** Yukun Guo, Wenqing Liu, Weiting Bu, Ruoxi Wang, Daoqing Su, Heng Li

**Affiliations:** 1 International Healthcare Center, Jinan Central Hospital Affiliated to Shandong First Medical University, Jinan, China; 2 Department of Neurosurgery, Jinan Central Hospital Affiliated to Shandong First Medical University, No. 5106 Jingshi Road, Licheng District, 250102 Jinan, China; 3 Center for Movement Disorders and Neuropathic Pain, BCI Patient Room, Xuanwu Jinan Hospital, Jinan, China; 4 Department of Neurology, Jinan Central Hospital Affiliated to Shandong First Medical University, Jinan, China; 5 Center for Neuroimmunology and Infection, Xuanwu Jinan Hospital, Jinan, China

**Keywords:** Parkinson’s disease, Inflammation, Motor impairment, Cerebrospinal fluid, Proteomics

## Abstract

**Purpose:**

Parkinson’s disease (PD) is a progressive neurodegenerative disorder in which neuroinflammation is recognized as a contributor to clinical progression. This study aimed to characterize the cerebrospinal fluid (CSF) inflammatory profile in mid- to late-stage PD patients and identify specific inflammatory proteins with potential clinical relevance to motor symptoms and disease severity.

**Methods:**

In this preliminary retrospective cross-sectional study, CSF samples were obtained from 25 patients with mid- to late-stage PD undergoing evaluation for deep brain stimulation (DBS) (mean disease duration: 10.24 ± 4.65 years) and 15 non-PD controls (essential tremor or dystonia) undergoing identical surgical procedures. The levels of 92 inflammation-related proteins were quantified using the Olink proximity extension assay (PEA). Based on the identified differentially expressed proteins (DEPs), we next performed preliminary exploratory comparisons of inflammatory profiles between the postural instability and gait difficulty (PIGD, *n* = 10) and tremor-dominant (TD, *n* = 10) PD subtypes. Additionally, preliminary correlation analyses were performed between the DEPs and Movement Disorder Society-Unified Parkinson’s Disease Rating Scale Part-III (MDS-UPDRS-III) scores to generate preliminary observations with limited clinical inference.

**Results:**

Using the Olink platform, 28 DEPs were identified between the PD and non-PD groups (*p* < 0.05). Subsequent protein-protein interaction network analysis identified IFN-γ as the central hub. Exploratory descriptive analyses of TD and PIGD subgroups are provided in Additional file 1: Supplementary Figure S1. Among all DEPs, IL-10RB (*r* = 0.440, 95% CI [0.054, 0.711], *p* = 0.028), CD8A (*r* = 0.415, 95% CI [0.024, 0.696], *p* = 0.039), and CXCL9 (*r* = 0.414, 95% CI [0.023, 0.696], *p* = 0.040) showed moderate correlations with MDS-UPDRS-III scores.

**Conclusion:**

In profiling 92 CSF inflammation-related proteins from 25 advanced PD patients (Hoehn–Yahr 3–4) and 11 controls, we identified 28 upregulated DEPs. IFN-γ emerged as a topologically connected hub in protein-protein interaction networks, and three proteins (IL-10RB, CD8A, CXCL9) showed moderate correlations with motor scores. As cross-sectional descriptive observations, these findings establish a preliminary framework to generate hypotheses for future mechanistic validation and biomarker discovery, while no functional causality can be inferred from the present results.

**Supplementary Information:**

The online version contains supplementary material available at https://doi.org/10.1186/s12883-026-05109-8.

## Introduction

Parkinson’s disease (PD) is a progressive neurodegenerative disease pathologically characterized by Lewy body accumulations, inflammation, and the loss of dopaminergic (DA) neurons in the brain. Accumulating evidence has established neuroinflammation plays a vital role in the pathophysiological process of PD [1]. Clinically, PD is a highly heterogeneous disorder with diverse motor and non-motor manifestations. These distinct clinical phenotypes are associated with different disease progression and prognoses. For instance, patients with the postural instability and gait difficulty (PIGD) subtype typically experience more severe motor and non-motor symptoms and have poorer prognoses than those with the tremor-dominant (TD) phenotype [2]. This clinical diversity likely reflects underlying pathophysiological differences. While the progression and subtyping of PD are primarily delineated based on motor symptom evaluation [3, 4], a potential association exists between neuroinflammation and impaired motor function in PD [5, 6].

Cerebrospinal fluid (CSF), given its anatomical proximity to the brain, serves as an ideal biofluid for investigating the pathophysiological process of PD [7]. However, due to the invasiveness of lumbar puncture, the application of CSF samples was less frequent in prior PD studies. A recent systematic review highlighted that among 26 CSF inflammatory factors of prior studies, only IL-1β has been consistently elevated in sporadic PD patients compared to healthy controls; changes in other key inflammatory markers such as IL-6, IL-8, and TNF-α remain inconclusive [8]. Thus, the comprehensive profiling of CSF inflammatory proteins is still needed to be clarified in mid- to late-stage PD.

Proximity extension assay (PEA) is a novel technology enabling high-throughput, multiplex protein biomarker analysis. Olink PEA has gradually become a new choice for proteomics, which is based on the high sensitivity and specificity to achieve accurate detection of proteins. Specifically, the Olink inflammation panel quantifies 92 target proteins associated with cellular response to cytokine stimulus, inflammatory response, apoptotic process, MAPK cascade, cell adhesion, regulation of immune response, and other inflammatory activities [9–11]. This panel has been applied in CSF studies of PD in early stage or atypical parkinsonisms [12–14]. Nevertheless, it remains unclear which inflammatory factors are most closely associated with disease progression, particularly in the mid- to-late stages of PD. This study aims to utilize the Olink inflammation panel to characterize the CSF inflammatory profile in mid- to late-stage PD and to evaluate the association between specific inflammation-related proteins and motor severity.

## Participants and methods

**Fig. 1:**
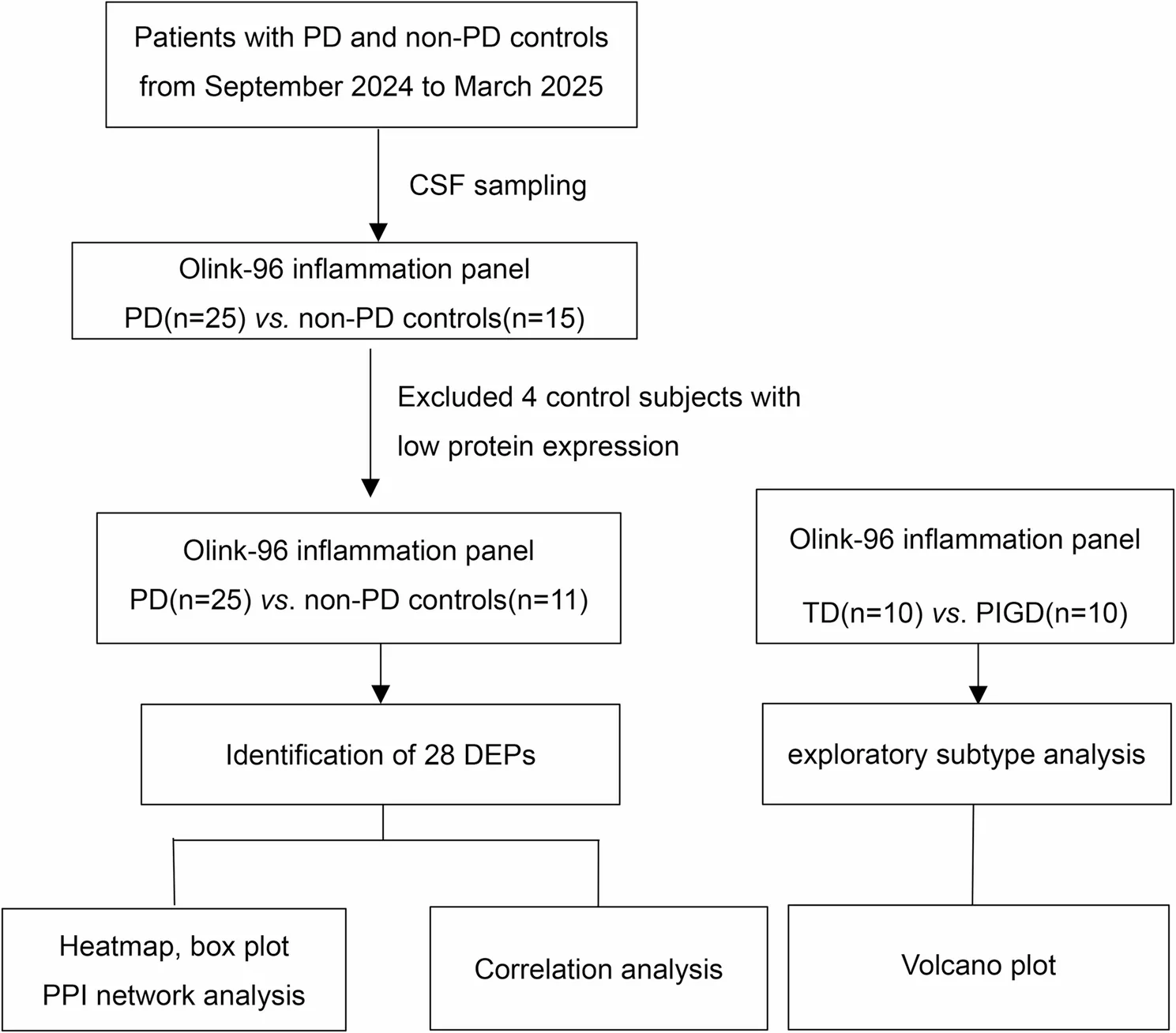
Flowchart of differentially expressed proteins (DEPs) identification. Abbreviations: CSF, cerebrospinal fluid; DEPs, differentially expressed proteins; PD, Parkinson’s disease; PIGD, postural instability and gait difficulty-dominant; PPI, protein-protein interaction; TD, tremor-dominant. Note: Differential protein analysis was not performed for the 5 indeterminate cases due to uncertain prognosis, as specified in the post-hoc protocol

Figure 1. Study workflow and bioinformatics analysis pipeline. CSF samples were collected from PD patients and non-PD controls, followed by Olink PEA profiling, differential expression analysis, and protein-protein interaction network construction.

### Collection of participant clinical information and CSF samples

All participants were recruited in this study at Jinan Central Hospital Affiliated to Shandong First Medical University between September 2024 and March 2025. The PD cohort included 25 patients who met the United Kingdom Parkinson’s Disease Society Brain Bank criteria and Hoehn-Yahr 3–4 stages of PD. Fifteen non-PD control subjects scheduled for surgical treatment were recruited based on the following inclusion criteria: (i) patients with essential tremor or primary focal dystonia; (ii) absence of infectious or autoimmune diseases; (iii) absence of dysarthria or psychiatric disorders affecting expression; iiii) absence of significant cognitive impairment or psychiatric symptoms. Based on the Olink detection results, four non-PD controls with significant global deviation from the cohort (intraclass correlation coefficient < 0.8, as identified by PCA) were subsequently excluded.

We collected the general demographic information of PD patients and non-PD controls, including gender, age, disease duration, age at onset, body mass index (BMI), presence or absence of diabetes mellitus (DM), and smoking status. For PD patients, the levodopa equivalent daily dose (LEDD) was calculated based on preoperative medication regimens. Motor function in PD patients was assessed during the “off” period using the Movement Disorder Society-Unified Parkinson’s Disease Rating Scale Part-III (MDS-UPDRS-III; Goetz et al., 2008) by two professional neurologists. The Hoehn and Yahr staging system was used to classify disease progression (stages 1-2.5: early stage; stages 3–5: mid-to late stage) [15]. Clinical motor subtypes were classified according to the Stebbins method [3]. Tremor and postural instability/gait dysfunction (PIGD) scores were derived from parts II and III of the MDS-UPDRS. Patients were categorized into three subtypes using the ratio method (tremor score/PIGD score): tremor-dominant (TD, ratio ≥ 1.5), PIGD-dominant (ratio ≤ 0.9), and indeterminate (ratio 0.9–1.5). Among the 25 PD patients, 10 were classified as PIGD, 10 as TD, and 5 as indeterminate. Consistent with prior studies [16], patients with the indeterminate subtype (*n* = 5) were excluded from subtype-stratified analyses, as this group represents a heterogeneous intermediate phenotype with poor longitudinal stability.

CSF samples were collected intraoperatively by dural puncture during deep brain stimulation (DBS) procedures. Sampling was performed immediately after dural opening and before electrode insertion and brain retraction, with approximately 1 mL of clear CSF obtained per participant. All specimens were visually inspected to exclude samples with blood contamination, discoloration, or hemolysis. Fresh CSF samples were centrifuged at 3000 rpm for 10 min at 4 °C immediately after collection. The clarified supernatants were aliquoted and stored at − 80 °C for subsequent proteomic analysis.

### Profiling of inflammation-related biomarkers

CSF samples from 25 PD patients and 15 non-PD controls were analyzed by using the Olink Proseek ^®^ Multiplex Inflammation panel (Olink Proteomics, Uppsala, Sweden) [10, 11], which targets 92 inflammation proteins (Additional file 1: Supplementary Table S1). The PEA technology involves oligonucleotide-labeled antibody pairs binding to their respective target proteins. When bound in proximity, the attached oligonucleotides hybridize and undergo DNA polymerization, generating a unique PCR reporter sequence. These sequences were detected, amplified, and quantified using a microfluidic real-time PCR instrument (Signature Q100, LC-Bio Technology CO., Ltd., Hangzhou, China). Assay results were reported as Normalized Protein Expression (NPX) values, followed by log2 transformation.

Quality control (QC) criteria were applied as follows: (1) For the entire assay plate, the internal QC standard deviation per sample was required to be < 0.2 NPX; (2) For individual samples, the deviation from the sample median was required to be < 0.3 NPX; samples exceeding this threshold were excluded. All 40 submitted samples passed QC successfully (100% pass rate). Protein levels were quantified as normalized protein expression (NPX) values using Olink NPX software. Proteins flagged by the software as below the limit of detection (LOD) were excluded from downstream analysis per the manufacturer’s standard guidelines. Using the Inflammation panel, 66 out of 92 target proteins met this criterion, yielding an average detection rate of 72%.

A comprehensive list of all inflammatory proteins is provided in Additional file 1: Supplementary Table S1.

### Statistical analysis

All statistical analyses were performed with SPSS 25.0 (IBM Corp., Armonk, NY, USA). Normality was assessed using the Shapiro–Wilk test (preferred for *n* < 50) supplemented by visual inspection of Q-Q plots. Variables with skewed distributions (Shapiro–Wilk *P* < 0.05 or clear visual deviation from normality) were analyzed using non-parametric tests (Mann–Whitney U); otherwise, parametric tests (t-test or ANOVA) were used. Pairwise associations were quantified using Pearson’s correlation coefficient for normally distributed variables and Spearman’s rank correlation coefficient for non-normally distributed variables. A two-tailed p-value < 0.05 was considered statistically significant.

Differentially expressed proteins (DEPs) between groups were identified by using Olink NPX Signature 3.0. Statistical significance was defined as *p* < 0.05. Intergroup comparisons (PD vs. non-PD controls; TD vs. PIGD) were conducted using the Welch two-sample t-test. Multiple testing correction was applied using the Benjamini-Hochberg method.

## Results

### Baseline characteristics

This study initially enrolled 40 participants. Of these, 25 CSF samples of patients with PD and 11 CSF samples of non-PD controls qualified for Olink protein detection. Baseline demographic and clinical characteristics are summarized in Table 1. The two groups showed broadly similar profiles with respect to sex, age, disease duration, age at onset, BMI, DM, and smoking status, though the limited sample size precludes definitive comparative inference. Among the 25 PD patients, 10 were classified as TD, 10 as PIGD, and 5 as indeterminate. Clinical features across these motor subtypes are outlined in Table 2; however, the small subgroup sizes limit robust statistical comparison and these data should be considered descriptive.

**Table 1:** Baseline characteristics of PD patients and non-PD controls

	PD	Non-PD	*P* Value
Sample Size	*N* = 25	*N* = 11	
Female, n (%)	17 (68.0)	7 (63.6)	1.000
Age (years)	60.08 ± 6.71	59.82 ± 10.5	0.941
Age at Onset (years)	49.84 ± 7.69	47.55 ± 11.13	0.478
Disease Duration (years)	10.0(7.0,13.5)	5.0(2.0,20.0)	0.202
BMI (kg/m^2^)	23.70 ± 4.34	24.48 ± 3.03	0.591
DM, n (%)	6 (24.0)	1 (9.1)	0.400
Smoking, n (%)	1 (4.0)	1 (9.1)	0.524

*Abbreviations*: *DM* Diabetes mellitus, *BMI* Body mass index, *PD* Parkinson’s disease, *PIGD* Postural instability and gait difficulty-dominant

**Table 2:** Comparison of clinical indicators among different motor subtypes of PD patients

	TD	PIGD	Indeterminate	*P* Value
Sample Size	*N* = 10	*N* = 10	*N* = 5	
Female, n (%)	5 (50.00)	6 (60.00)	5 (100.00)	0.254
Age (years)	57.70 ± 8.22	60.70 ± 3.62	63.60 ± 7.64	0.267
Age at Onset (years)	46.50 ± 8.53	51.80 ± 4.98	52.60 ± 9.40	0.209
Disease Duration (years)	11.20 ± 5.94	8.90 ± 3.60	11.00 ± 3.67	0.518
MDS-UPDRS III (scores)	63.40 ± 23.34	48.10 ± 7.00	66.40 ± 22.83	0.102
LEDD (mg/day)	827.03 ± 358.88	885.15 ± 196.85	672.50 ± 233.59	0.396

*Abbreviations*: *LEDD* Levodopa equivalent daily dose, *MDS-UPDRS-III* Movement Disorder Society-Unified Parkinson's Disease Rating Scale Part-III

### DEPs in PD vs. non-PD groups

**Fig. 2:**
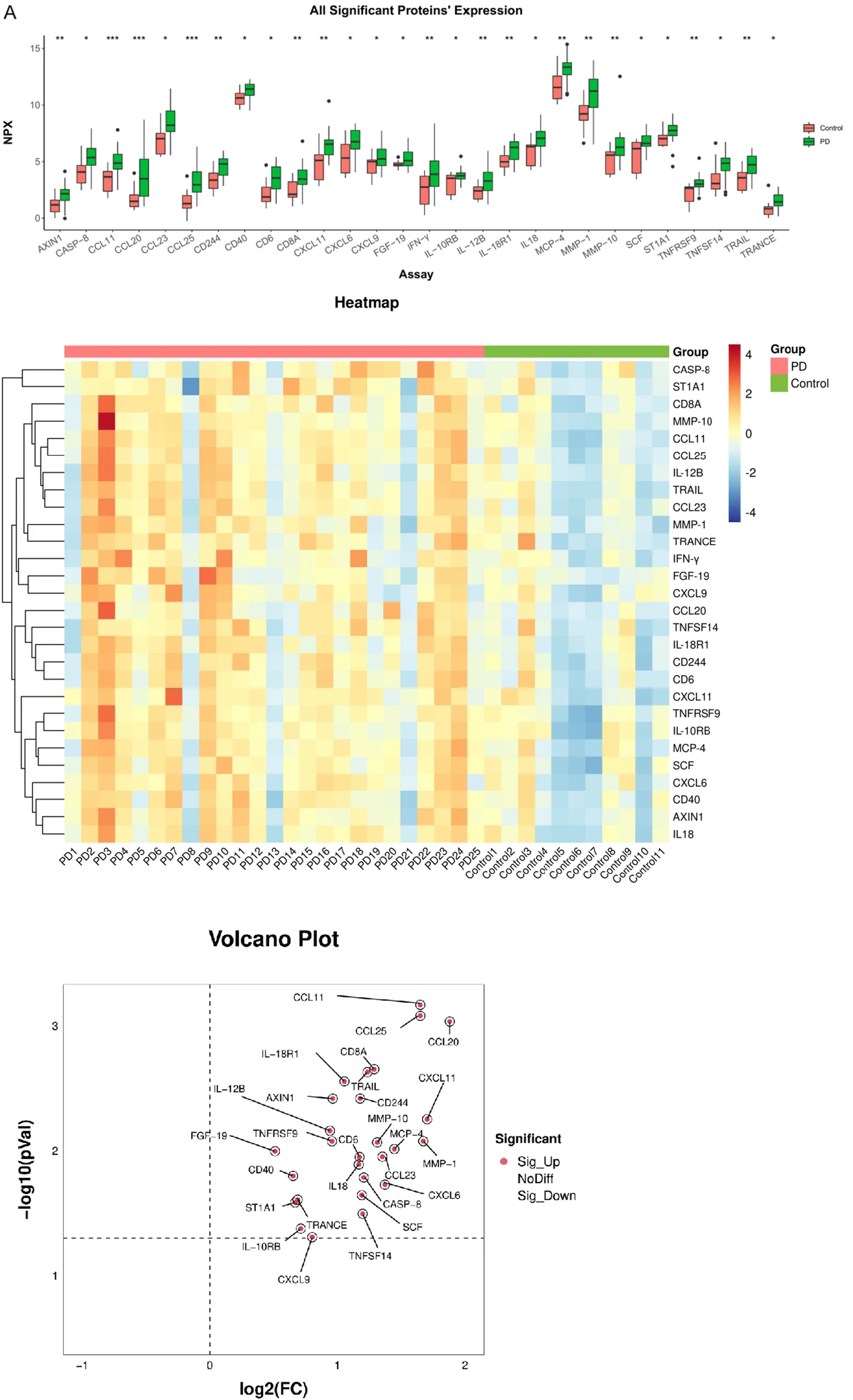
Differentially expressed protein levels between patients with PD and non-PD control subjects. **A **Box plot of 28 DEPs. **B** Heatmap of 28 DEPs. Significance levels: *P < 0.05, **P < 0.01, ***P < 0.001. **C **Volcano plot of 28 DEPs. The gray dashed line indicates an exploratory cutoff of *p*-value < 0.05. Red dots mark significantly upregulated proteins passing the exploratory threshold

Analysis of 92 inflammation-related proteins identified 28 DEPs between the PD group and the non-PD control group. All 28 DEPs were significantly upregulated in the PD group, including CCL11, CCL25, CCL20, CD8A, TRAIL, IL-18R1, CD244, AXIN1, IFN-γ, CXCL11, IL-12B, MMP-1, TNFR3F9, MMP-10, MCP-4, FGF-19, CCL23, CD6, IL18, CD40, CASP-8, CXCL6, SCF, TRANCE, ST1A1, TNFSF-14, IL-10RB, CXCL9 (Fig. 2A). The heatmap and volcano plot further illustrated the expression of DEPs across groups (Fig. 2B and C).

### Enrichment analysis and PPI in PD vs. non-PD groups

**Fig. 3:**
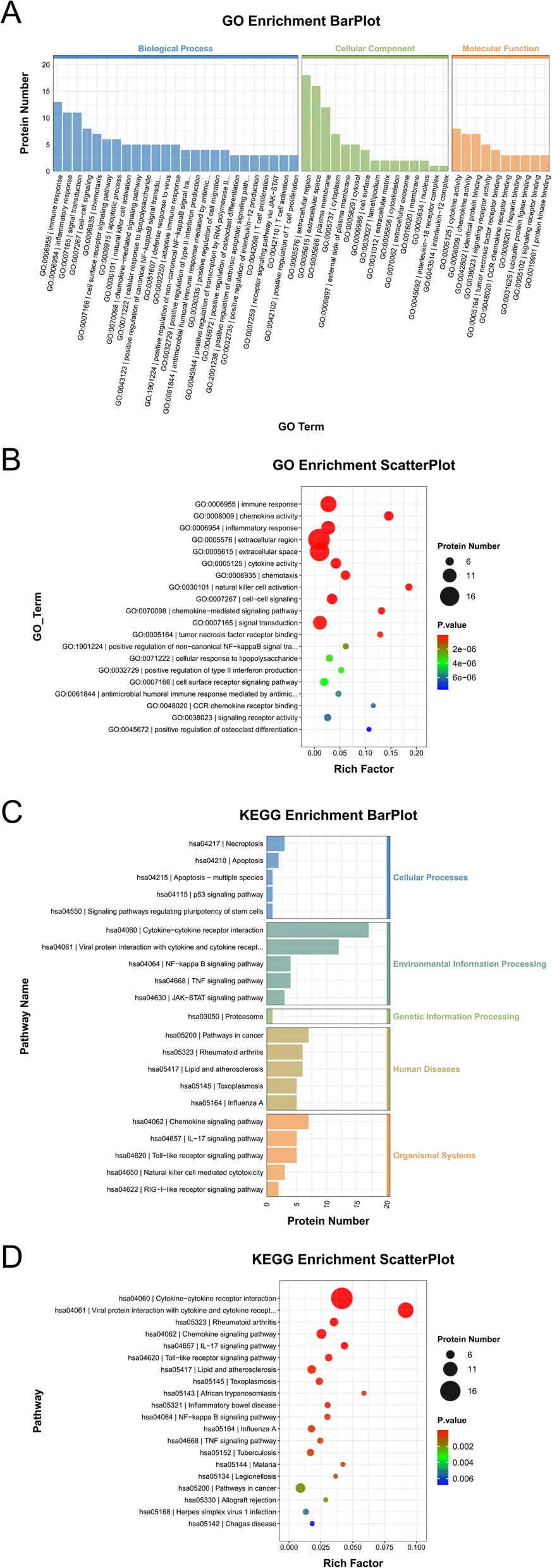
GO and KEGG enrichment analysis of DEPs between PD and non-PD controls. **A** GO enrichment analysis showing the top enriched biological process, cellular component, and molecular function categories. **B** Bubble plot of GO enrichment; bubble color indicates the significance of enriched GO terms (*P* value), and bubble size corresponds to the enrichment factor. **C** KEGG pathway enrichment analysis; the ordinate shows significantly enriched pathways, and the abscissa indicates the number of enriched proteins (rich factor ≤ 1). **D** Bubble plot of KEGG enrichment; bubble color indicates pathway significance, and the enrichment factor represents the proportion of DEPs mapped to each pathway relative to all identified proteins

Based on the differentially expressed inflammatory proteins identified in CSF between PD patients and non-PD controls, we conducted Gene Ontology (GO) and Kyoto Encyclopedia of Genes and Genomes (KEGG) pathway enrichment analyses. GO enrichment analysis revealed that genes corresponding to differentially expressed proteins were significantly enriched in biological processes related to immune response, inflammatory response, and signal transduction. Cellular Component (CC) was mainly enriched in extracellular space, extracellular region and plasma membrane. The Molecular Function (MF) category was mainly enriched in cytokine activity, protein binding and chemokine activity (Fig. 3A and B). KEGG enrichment analysis revealed a marked enrichment of differentially expressed proteins in immune-related pathways, with “Cytokine–cytokine receptor interaction” and “Viral protein interaction with cytokine and cytokine receptor” representing the top-ranking pathways (Fig. 3C and D).

**Fig. 4:**
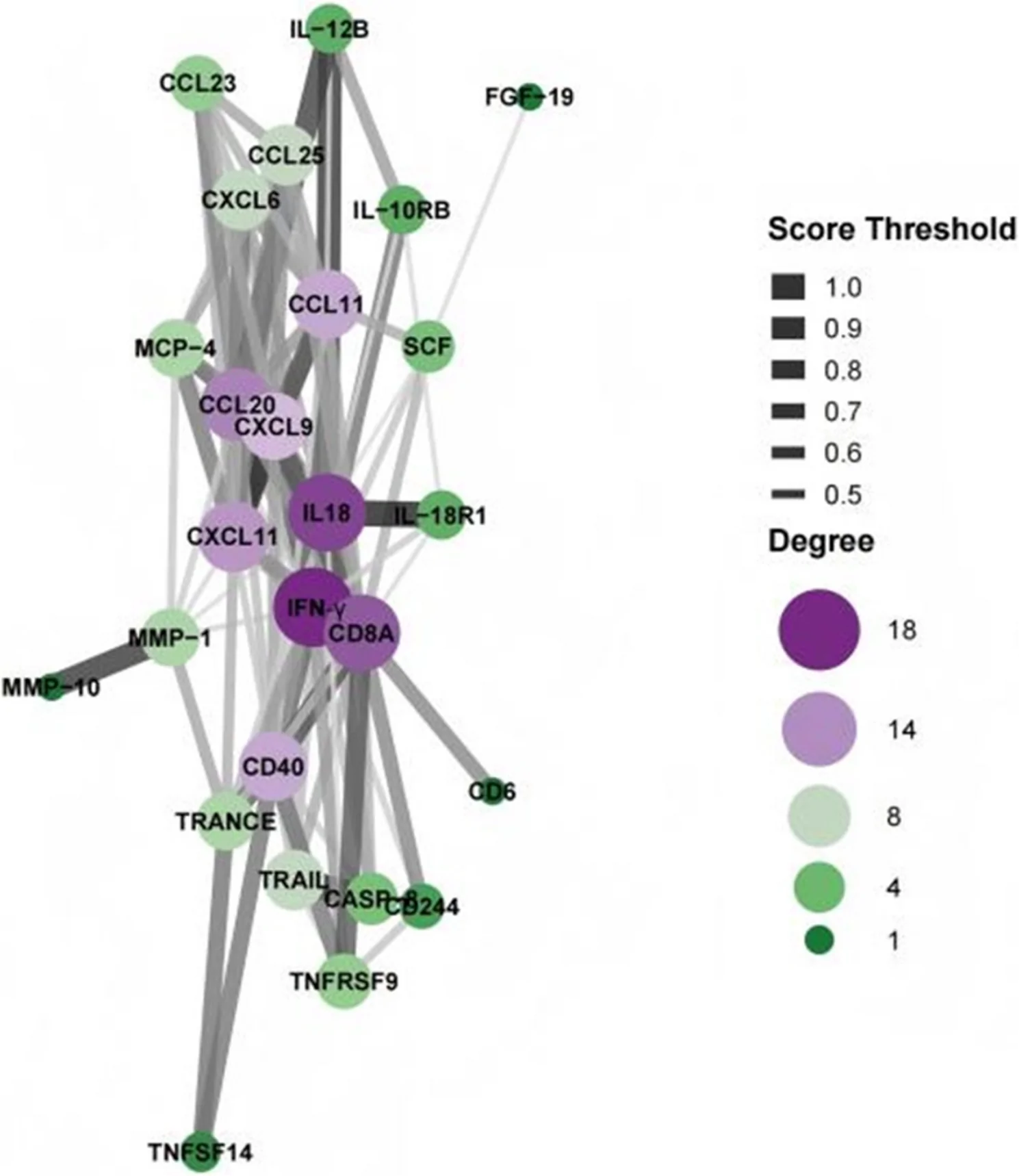
The protein-protein interaction network visualizes potential interactions between a group of proteins. PPI network analysis was performed using the String DB database (http://string-db.org/) based on 28 DEPs identified by Olink 92-plex Inflammation panel. Network topology of DEPs. Nodes represent individual proteins, with node size and color intensity proportional to the degree of connectivity (degree): larger and darker purple nodes indicate higher degree values, whereas smaller and lighter green nodes indicate lower degree values. Edges represent predicted protein–protein interactions, with line thickness and grayscale corresponding to the STRING confidence score (score threshold: 0.5–1.0); thicker and darker edges denote higher interaction confidence

Correlation analysis was performed on the 28 DEPs, and a protein-protein interaction (PPI) network was subsequently constructed using the STRING database. IFN-γ was identified as a key hub node within this network, suggesting that IFN-γ and its interacting partners may play a critical role in the pathogenesis of PD (Fig. 4).

### DEPs in TD vs. PIGD subtypes

Given the small subgroup sizes, we performed exploratory descriptive comparisons of TD (*n* = 10) and PIGD (*n* = 10) subtypes; these results are presented in Additional file 1: Supplementary Figure S1 without formal inferential statistics.

### Correlation between inflammatory protein levels and motor severity in PD

**Fig. 5:**
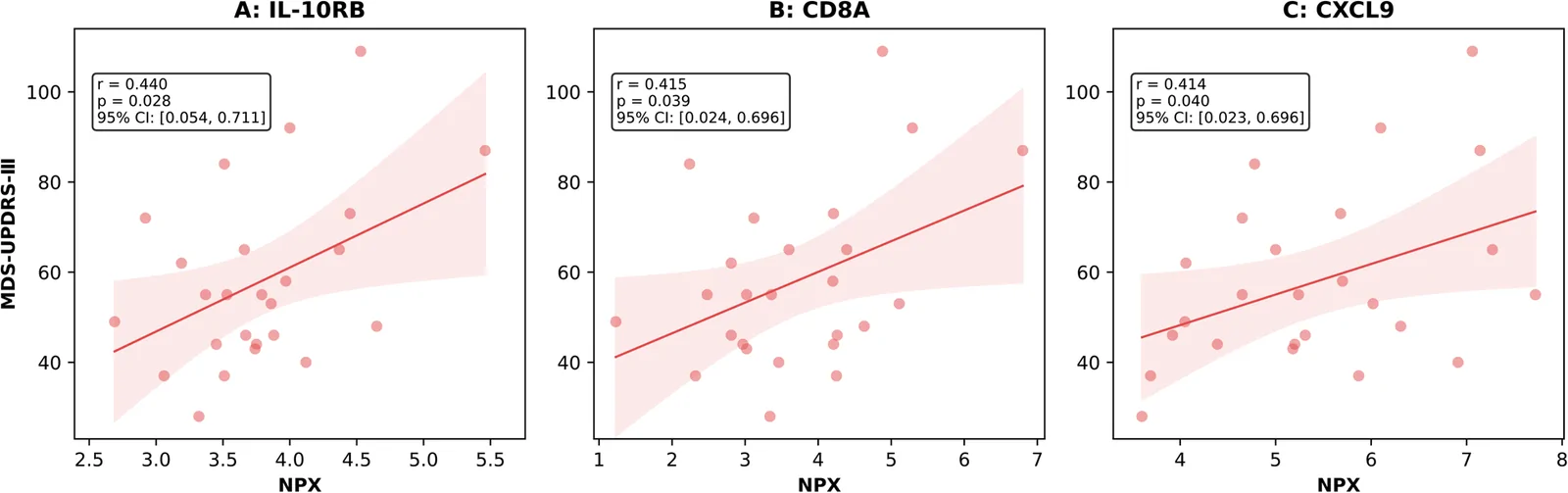
Scatter plots of correlation between DEPs and MDS-UPDRS-Ⅲ scores. **A** IL-10RB (r = 0.440, *p* = 0.028). **B** CD8A (r = 0.415, *p* = 0.039). **C** CXCL9 (r = 0.414, *p* = 0.040).Scatter plots showing the relationship between cerebrospinal fluid protein levels (NPX, normalized protein expression) and motor symptom severity as assessed by the Movement Disorder Society–Unified Parkinson's Disease Rating Scale Part-III (MDS-UPDRS-III). **A** IL-10RB (r = 0.440, 95% CI [0.054, 0.711], *p* = 0.028). **B** CD8A (r = 0.415, 95% CI [0.024, 0.696], *p* = 0.039). **C** CXCL9 (r = 0.414, 95% CI [0.023, 0.696], *p* = 0.040). P-values represent nominal significance without multiple-comparison correction and should be interpreted as exploratory. Each data point represents an individual PD patient. The red line indicates the linear regression fit. All three proteins showed moderate positive correlations with MDS-UPDRS-III scores. NPX, normalized protein expression; MDS-UPDRS-III, Movement Disorder Society–Unified Parkinson's Disease Rating Scale Part-III

To investigate potential associations between protein expression and motor symptom severity, we performed correlation analyses between DEP levels and MDS-UPDRS-III scores. Modest correlations were observed between MDS-UPDRS-III scores and IL-10RB (*r* = 0.440, 95% CI [0.054, 0.711], *p* = 0.028), CD8A (*r* = 0.415, 95% CI [0.024, 0.696], *p* = 0.039), and CXCL9 (*r* = 0.414, 95% CI [0.023, 0.696], *p* = 0.040) (Fig. 5).

## Discussion

We utilized Olink technology to analyze CSF samples from mid- to late-stage PD and non-PD patients. Among the 92 inflammation-related proteins, 28 DEPs were identified between the PD and non-PD groups. Subsequent PPI network analysis pinpointed IFN-γ as the key hub protein. Preliminary exploratory descriptive comparisons of inflammatory profiles between PIGD and TD subgroups are provided in Additional file 1: Supplementary Figure S1. Additionally, three proteins (IL-10RB, CD8A, and CXCL-9) showed preliminary correlation with MDS-UPDRS-III scores.

Of the 28 DEPs, five inflammatory proteins (MMP-10, CCL23, IL-18R, CD8A, and FGF19) have been previously reported in CSF proteomic studies of PD patients [12–14]. Other factors such as TNFSF9 and CCL23 were also documented in plasma proteomic investigations [17]. Our study revealed a greater number of DEPs compared to previous CSF proteomic studies, which may be attributed to differences in cohort characteristics [12–14]. Specifically, our PD cohort exhibited longer disease durations and higher motor dysfunction scores, suggesting more advanced disease severity and potentially more pronounced neuroinflammation. Additionally, CSF samples were collected via cerebral dural puncture immediately after dural opening and prior to brain retraction. This methodological approach may enhance the detection of inflammatory proteins, thereby providing a more accurate reflection of cerebral inflammation [18]. However, brain-proximal sampling may yield higher protein concentrations than lumbar puncture (LP) owing to rostrocaudal gradients, limiting direct comparability with prior LP-based studies.

The limited overlap between our differentially expressed proteins (DEPs) and those from previous large-scale studies likely reflects three key distinctions: (i) our use of CSF versus plasma/serum, given the sparse blood-CSF correlation of inflammatory profiles in PD [8]; (ii) our cohort’s advanced disease stage (H&Y 3–4) compared to broader-stage populations in other studies; and (iii) technical differences in proteomic platforms and pipelines. These factors suggest our findings capture a distinct compartment- and stage-specific biological landscape of advanced PD, rather than contradicting prior report.

GO enrichment analysis revealed that these differentially expressed proteins were significantly enriched in biological processes including immune response, inflammatory response, and signal transduction; cellular components such as extracellular space, extracellular region, and plasma membrane; and molecular functions including cytokine activity, protein binding, and chemokine activity. KEGG pathway enrichment analysis revealed that the differentially expressed proteins were predominantly concentrated in “Cytokine–cytokine receptor interaction” and “Viral protein interaction with cytokine and cytokine receptor”. These pathways are not isolated entities; they frequently co-enrich with and functionally intertwine with the chemokine signaling pathway, TNF signaling pathway, NF-κB signaling pathway, and JAK-STAT signaling pathway [19]. This coordinated enrichment pattern suggests that the neuroinflammatory process in PD, as reflected in craniotomy-derived CSF, involves a broad network of intercellular communication rather than isolated pathway dysregulation.

PPI analysis generated a conceptual network designating IFN-γ as the topologically central protein among the DEPs. IFN-γ, primarily secreted by activated T cells, has been demonstrated to promote inflammatory activation of astrocytes and microglia in PD animal models [20]. Among the DEPs, IL-18, IL-12, and CXCL-9 contribute to recruiting Th1 and CD8 + T cells to secrete IFN-γ [21–24], yet mechanistic evidence is largely limited to animal models. Moreover, although IFN-γ synergizes with inflammatory factors such as TNF-α, IL-18, and SCF to promote T-cell maturation and activation [25–27], supporting data remain confined to animal studies or plasma protein levels of IL-18 and TNF-α in specific disease contexts. These activated T cells, in turn, release inflammatory factors like CXCL-11, CXCL-9, and IFN-γ [28]. Collectively, the available evidence identifies IFN-γ, by network topology alone, as a highly connected node within this DEP network.

Accumulating evidence indicates that PD is a heterogeneous neurodegenerative disorder characterized by diverse clinicopathological phenotypes. Generally, the TD subtype is associated with a more benign disease course and slower progression, indicating a better prognosis than the PIGD subtype [29]. An exploratory analysis of DEP levels by motor subtype (PIGD vs. TD) revealed preliminary numerical differences between groups; however, the small subgroup sample size (*n* = 10 per group) precludes reliable statistical inference. The data presented in Additional file 1: Supplementary Figure S1 are purely descriptive and should not be interpreted as indicating differential inflammatory profiles. Future studies with larger cohorts are warranted to investigate potential subtype-specific neuroinflammatory patterns.

We also observed preliminary evidence for positive correlations between MDS-UPDRS-III scores and the inflammatory proteins IL-10RB, CD8A, and CXCL9. IL-10RB serves as a receptor for both IL-10 and IFN-γ. Although IL-10 is generally considered an anti-inflammatory cytokine, elevated peripheral IL-10 levels in the peripheral blood of PD patients have been reported, suggesting that the significant upregulation of IL-10RB may represent a compensatory protective mechanism during the disease progression [30]. We observed that the elevated CSF CD8A level correlated positively with motor symptom severity in PD patients. This finding is consistent with a potential involvement of CD8 + T-cell infiltration in the neuroinflammatory milieu of PD [31], though causality cannot be inferred from these cross-sectional data. The prior study has demonstrated IFN-γ-induced T cell infiltration can upregulate the level of CXCL9 expression in cerebral astrocytes and microvascular endothelial cells under the autoimmune conditions [32]. The elevated CXCL9 level detected in our PD cohort parallels reports of T-cell-mediated inflammation in PD, providing a hypothetical link that requires mechanistic validation.

Our study has several shortcomings. The sample size was relatively modest, so multivariate modeling was not really feasible; including covariates such as disease duration, LEDD, age, or sex would have risked overfitting and unstable estimates. We cannot rule out that the observed motor score correlations partly reflect confounding by disease severity or medication effects rather than independent biological signals. Still, all recruited PD patients were in the mid- to late-stage disease with consistently good responses to PD medications, which makes atypical PD or PD-plus syndrome unlikely. We did not have DaTscan or post-mortem pathological confirmation—an obvious weakness. That said, the stringent clinical diagnostic process and long-term follow-up provide reasonable safeguards against misclassification. Finally, although cerebral dural puncture may yield CSF samples closer to the CNS inflammatory milieu than lumbar puncture, craniotomy itself introduces potential confounding. Surgical trauma, meningeal disruption, and intraoperative physiological changes can all alter protein levels independently, so attributing the observed inflammatory profile solely to PD pathophysiology would be premature.

The control group introduces another layer of complexity. Rather than healthy individuals, our controls were patients with essential tremor or primary focal dystonia. Primary dystonia typically presents as a benign, slowly progressive motor disorder and is generally considered as a cerebral microstructural defect rather than a neurodegenerative disease [33, 34]. We cannot fully discount subtle neuroinflammatory changes in these conditions, however. Whether the inflammatory signature we identified is truly PD-specific would require comparison with healthy controls and other neurodegenerative diseases.

Beyond these issues, the study design and assay constraints deserve mention. This was a retrospective study, so causal claims remain tenuous and the inflammatory profile we describe awaits replication in a prospective setting. The assay panel itself was also a constraint: we were bound by its commercial coverage, had no IL-1β data, and had to drop proteins that failed QC. The profile is therefore almost certainly incomplete. Medication differences are another unresolved issue. Dopaminergic drugs are thought to exert only modest immunomodulatory effects, but modest is not zero, and their confounding influence cannot be purged entirely. A preoperative washout of at least 12 h likely blunted acute drug effects, yet chronic exposure may have left a longer-lasting immunological imprint—perhaps subtly shifting pro-inflammatory cytokine levels in ways we cannot fully capture. The control patients, meanwhile, were on entirely different agents, which only muddies the comparison further. How much of this inflammatory signal is intrinsic to PD pathophysiology rather than shaped by chronic medication exposure remains unclear. Disentangling the two would require looking at drug-naïve or newly diagnosed patients, something the present study cannot do.

## Conclusion

In summary, using Olink PEA proteomics, we quantified 92 inflammation-related proteins in CSF samples from 25 mid- to late-stage PD patients and 11 non-PD controls and identified 28 upregulated DEPs. Network analysis positioned IFN-γ as a topologically central node within the inflammatory protein network, and three DEPs showed moderate correlations with motor dysfunction scores. These descriptive, cross-sectional findings provide a hypothesis-generating basis for prioritizing inflammatory pathway candidates in future mechanistic studies and for exploring progression biomarkers, pending functional validation. 

## Supplementary Information

Supplementary Material 1.

Supplementary Material 2.

## Data Availability

The authors confirm that the data supporting the findings of this study are available within the article and its supplementary materials.
