# Supplementary material for: A preliminary analysis of the inflammatory protein landscape in the CSF of mid- to late-stage Parkinson’s disease: associations with motor severity and subtypes
Source: BMC Neurol. 2026 Jul 1;26:595. doi: 10.1186/s12883-026-05109-8 (PMC13591893; doi:10.1186/s12883-026-05109-8)
Supplement: Supplementary file 1 [file 12883_2026_5109_MOESM1_ESM.docx]

**Table of Contents:**

**Table S1:** The information of 92 inflammation-related proteins in Olink analysis.

The raw data of the Olink proteomics analyisis (XLSX)

**Table S1.** Detailed information of 92 inflammation-related biomarkers

| OlinkID | UniProt | Assay | Description |
| --- | --- | --- | --- |
| OID00471 | P10145 | IL8 | Interleukin-8 |
| OID00472 | P15692 | VEGFA | Vascular endothelial growth factor |
| OID05124 | P01732 | CD8A | T-cell surface glycoprotein CD8 alpha chain |
| OID00474 | P80098 | MCP-3 | C-C motif chemokine 7 |
| OID00475 | P39905 | GDNF | Glial cell line-derived neurotrophic factor |
| OID00476 | Q9H5V8 | CDCP1 | CUB domain-containing protein 1 |
| OID00477 | Q9BZW8 | CD244 | Natural killer cell receptor 2B4 |
| OID00478 | P13232 | IL7 | Interleukin-7 |
| OID00479 | O00300 | OPG | Tumor necrosis factor receptor superfamily member 11B |
| OID00480 | P01137 | LAP TGF-beta-1 | Transforming growth factor beta-1 |
| OID00481 | P00749 | uPA | Urokinase-type plasminogen activator |
| OID00482 | P05231 | IL6 | Interleukin-6 |
| OID00483 | Q9P0M4 | IL-17C | Interleukin-17C |
| OID00484 | P13500 | MCP-1 | C-C motif chemokine 2 |
| OID00485 | Q16552 | IL-17A | Interleukin-17A |
| OID00486 | O14625 | CXCL11 | C-X-C motif chemokine 11 |
| OID00487 | O15169 | AXIN1 | Axin-1 |
| OID00488 | P50591 | TRAIL | Tumor necrosis factor ligand superfamily member 10 |
| OID00489 | Q9UHF4 | IL-20RA | Interleukin-20 receptor subunit alpha |
| OID00490 | Q07325 | CXCL9 | C-X-C motif chemokine 9 |
| OID00491 | P28325 | CST5 | Cystatin-D |
| OID00492 | P14784 | IL-2RB | Interleukin-2 receptor subunit beta |
| OID00493 | P01583 | IL-1 alpha | Interleukin-1 alpha |
| OID00494 | P13725 | OSM | Oncostatin-M |
| OID00495 | P60568 | IL2 | Interleukin-2 |
| OID00496 | P09341 | CXCL1 | Growth-regulated alpha protein |
| OID00497 | Q969D9 | TSLP | Thymic stromal lymphopoietin |
| OID00498 | P13236 | CCL4 | C-C motif chemokine 4 |
| OID00499 | P30203 | CD6 | T-cell differentiation antigen CD6 |
| OID00500 | P21583 | SCF | Kit ligand |
| OID00501 | Q14116 | IL18 | Interleukin-18 |
| OID00502 | Q13291 | SLAMF1 | Signaling lymphocytic activation molecule |
| OID00503 | P01135 | TGF-alpha | Protransforming growth factor alpha |
| OID00504 | Q99616 | MCP-4 | C-C motif chemokine 13 |
| OID00505 | P51671 | CCL11 | Eotaxin |
| OID00506 | O43557 | TNFSF14 | Tumor necrosis factor ligand superfamily member 14 |
| OID00507 | Q9GZV9 | FGF-23 | Fibroblast growth factor 23 |
| OID00508 | Q13651 | IL-10RA | Interleukin-10 receptor subunit alpha |
| OID00509 | P12034 | FGF-5 | Fibroblast growth factor 5 |
| OID00510 | P03956 | MMP-1 | Interstitial collagenase |
| OID00511 | P42702 | LIF-R | Leukemia inhibitory factor receptor |
| OID00512 | Q9NSA1 | FGF-21 | Fibroblast growth factor 21 |
| OID00513 | Q99731 | CCL19 | C-C motif chemokine 19 |
| OID00514 | Q13261 | IL-15RA | Interleukin-15 receptor subunit alpha |
| OID00515 | Q08334 | IL-10RB | Interleukin-10 receptor subunit beta |
| OID00516 | Q8N6P7 | IL-22 RA1 | Interleukin-22 receptor subunit alpha-1 |
| OID00517 | Q13478 | IL-18R1 | Interleukin-18 receptor 1 |
| OID00518 | Q9NZQ7 | PD-L1 | Programmed cell death 1 ligand 1 |
| OID00519 | P01138 | Beta-NGF | Beta-nerve growth factor |
| OID00520 | P42830 | CXCL5 | C-X-C motif chemokine 5 |
| OID00521 | O14788 | TRANCE | Tumor necrosis factor ligand superfamily member 11 |
| OID00522 | P14210 | HGF | Hepatocyte growth factor |
| OID00523 | P29460 | IL-12B | Interleukin-12 subunit beta |
| OID00524 | Q13007 | IL-24 | Interleukin-24 |
| OID00525 | P35225 | IL13 | Interleukin-13 |
| OID00526 | Q5T4W7 | ARTN | Artemin |
| OID00527 | P09238 | MMP-10 | Stromelysin-2 |
| OID00528 | P22301 | IL10 | Interleukin-10 |
| OID05548 | P01375 | TNF | Tumor necrosis factor |
| OID00530 | P55773 | CCL23 | C-C motif chemokine 23 |
| OID00531 | P06127 | CD5 | T-cell surface glycoprotein CD5 |
| OID00532 | P10147 | CCL3 | C-C motif chemokine 3 |
| OID00533 | P49771 | Flt3L | Fms-related tyrosine kinase 3 ligand |
| OID00534 | P80162 | CXCL6 | C-X-C motif chemokine 6 |
| OID00535 | P02778 | CXCL10 | C-X-C motif chemokine 10 |
| OID00536 | Q13541 | 4E-BP1 | Eukaryotic translation initiation factor 4E-binding protein 1 |
| OID00537 | Q9NYY1 | IL-20 | Interleukin-20 |
| OID00538 | Q8IXJ6 | SIRT2 | NAD-dependent protein deacetylase sirtuin-2 |
| OID00539 | Q9NRJ3 | CCL28 | C-C motif chemokine 28 |
| OID01213 | Q8NFT8 | DNER | Delta and Notch-like epidermal growth factor-related receptor |
| OID00541 | P80511 | EN-RAGE | Protein S100-A12 |
| OID00542 | P25942 | CD40 | Tumor necrosis factor receptor superfamily member 5 |
| OID00543 | O95760 | IL33 | Interleukin-33 |
| OID05547 | P01579 | IFN-γ | Interferon gamma |
| OID00545 | O95750 | FGF-19 | Fibroblast growth factor 19 |
| OID00546 | P05112 | IL4 | Interleukin-4 |
| OID00547 | P15018 | LIF | Leukemia inhibitory factor |
| OID00548 | Q99748 | NRTN | Neurturin |
| OID00549 | P80075 | MCP-2 | C-C motif chemokine 8 |
| OID00550 | Q14790 | CASP-8 | Caspase-8 |
| OID00551 | O15444 | CCL25 | C-C motif chemokine 25 |
| OID00552 | P78423 | CX3CL1 | Fractalkine |
| OID00553 | Q07011 | TNFRSF9 | Tumor necrosis factor receptor superfamily member 9 |
| OID00554 | P20783 | NT-3 | Neurotrophin-3 |
| OID00555 | O43508 | TWEAK | Tumor necrosis factor ligand superfamily member 12 |
| OID00556 | P78556 | CCL20 | C-C motif chemokine 20 |
| OID00557 | P50225 | ST1A1 | Sulfotransferase 1A1 |
| OID00558 | O95630 | STAMBP | STAM-binding protein |
| OID00559 | P05113 | IL5 | Interleukin-5 |
| OID00560 | P00813 | ADA | Adenosine deaminase |
| OID00561 | P01374 | TNFB | Lymphotoxin-alpha |
| OID00562 | P09603 | CSF-1 | Macrophage colony-stimulating factor 1 |
